# The need to compare: assessing the level of agreement of three high-throughput assays against *Plasmodium falciparum* mature gametocytes

**DOI:** 10.1038/srep45992

**Published:** 2017-04-05

**Authors:** Leonardo Lucantoni, Sasdekumar Loganathan, Vicky M. Avery

**Affiliations:** 1 Discovery Biology, Griffith Institute for Drug Discovery, Griffith University, 4111 Nathan, Queensland, Australia

## Abstract

Whole-cell High-Throughput Screening (HTS) is a key tool for the discovery of much needed malaria transmission blocking drugs. Discrepancies in the reported outcomes from various HTS *Plasmodium falciparum* gametocytocidal assays hinder the direct comparison of data and ultimately the interpretation of the transmission blocking potential of hits. To dissect the underlying determinants of such discrepancies and assess the impact that assay-specific factors have on transmission-blocking predictivity, a 39-compound subset from the Medicines for Malaria Venture Malaria Box was tested in parallel against three distinct mature stage gametocytocidal assays, under strictly controlled parasitological, chemical, temporal and analytical conditions resembling the standard membrane feeding assay (SMFA). Apart from a few assay-specific outliers, which highlighted the value of utilizing multiple complementary approaches, good agreement was observed (average ΔpIC_50_ of 0.12 ± 0.01). Longer compound incubation times improved the ability of the least sensitive assay to detect actives by 2-fold. Finally, combining the number of actives identified by any single assay with those obtained at longer incubation times yielded greatly improved outcomes and agreement with SMFA. Screening compounds using extended incubation times and using multiple *in vitro* assay technologies are valid approaches for the efficient identification of biologically relevant malaria transmission blocking hits.

During the past three decades, therapeutic and vector control efforts have resulted in an encouraging 60% global decrease in the malaria mortality rate^1^. However, half the world population remains at risk of malaria, with 90% of the nearly half a million annual malaria-related deaths occurring in Africa, where the predominant species is *Plasmodium falciparum*^1^. To achieve the ambitious goal of malaria eradication, more needs to be done to further reduce mortality^2^,^3^. Unfortunately, the increasing drug and insecticide resistance in parasite^4^ and vector^5^ populations, combined with delays in developing new drugs to replace the current first line treatment (Artemisinin Combination Therapy, ACT), might not only prevent eradication being achieved but may even lead to a resurgence of malaria^6^. Hence, new drugs with new targets are desperately needed.

The difficulties associated with therapeutic control of this parasitic disease are due to its complex life cycle involving two different hosts with multiple proliferative and non-proliferative stages. Blocking *P. falciparum* transmission is deemed necessary for the eradication goal to succeed^7^. The continual transmission of malaria relies on gametocytes, the sexual stage of the parasite^8^, being taken up by the mosquito vector, thereby enabling the continuation of the parasite lifecycle. Gametocytes themselves do not replicate, they rather undergo a process called gametocytogenesis, which incorporates differentiation through five morphologically distinct maturation stages, named I to V, requiring a period of 10–14 days^9^. Mature, crescent-shaped stage V gametocytes are sexually dimorphic and are taken up with other parasitic stages and blood products by Anopheline mosquitoes during a blood meal from an infected individual^10^. Only the mature gametocyte stage survives in the mosquito gut and is able to undergo sexual replication and ultimately produce new infective sporozoites. Subsequent feeding of infected mosquitoes on humans results in the transmission of sporozoites and ultimately, following further asexual replication, malaria.

Gametocytes are less sensitive to most clinically used antimalarial drugs^11^, thus the drugs used to treat malaria have limited impact on transmission. Current drug discovery efforts focus on the identification of antimalarial compounds, which not only are highly efficacious in treating the clinical stages, but are also effective across multiple life cycle stages, including gametocytes.

Phenotypic assays have been the tool of choice for the screening of large chemical libraries for malaria drug discovery efforts in recent years^12^,^13^. Such assays are not reliant on prior knowledge of the molecular target, thus allowing the simultaneous identification of compounds with multiple modes of action and novel targets. The use of whole cell-based assays thus simplifies the transmission-blocking drug discovery process in the current context of limited knowledge of gametocyte biology and urgent need to find novel targets^14^,^15^. In addition, compounds active in whole parasite assays are assumed to effectively penetrate the parasite cellular membranes to exert a measurable activity, thus whole organism assays provide a valuable basis for drug discovery by more closely mimicking the *in vivo* situation than target-based assays^16^,^17^.

Since 2012, multiple gametocytocidal screening assays with variable throughput have been developed by numerous research groups, resulting in a variety of assay designs and approaches covering the whole gametocytogenesis and gametogenesis processes. These include the use of the metabolic indicator AlamarBlue^18^, parasite lactate dehydrogenase (pLDH)^19^, ATP production^20^,^21^, expression of reporter genes in transgenic parasites lines^22^,^23^,^24^,^25^, fluorescent staining of gametocytes^26^, fluorescent staining of female gametes^27^, detection of female gamete specific antigens^28^,^29^,^30^ and time-course imaging of exflagellating male gametocytes^28^.

Independent approaches were taken by the various laboratories for the development of their assays and this often resulted in a wide range of settings adopted for core assay parameters. These were previously reviewed by our group^31^, and include: (1) laboratory-related variables such as culturing method, media composition, compound handling and dispensing equipment, (2) assay-related variables such as parasite life cycle stage assessed, time of incubation with compounds and with detection reagents, screening compound concentration, type of controls, technology used for readout, and (3) data analysis-related variables such as normalization approach, hit activity cut-off selection, number of concentrations used for dose-response assays, curve fitting constraints and criteria for acceptability of the IC_50_ values.

Due to the above issues, the direct comparison of different assays has been impossible, leaving unanswered the question of which are the factors that most contribute to assay outcomes and how these impact the predictivity of transmission blocking potential by an assay. Importantly, the contributing role of the technology used has been particularly difficult to ascertain. The hypothesis underlying this work is that once the laboratory-related and analysis-related core variability sources have been corrected for, the contribution given by assay-related factors can be dissected to understand the impact each factor has on the assay outcomes.

For this purpose a useful tool is offered by the Medicines for Malaria Venture (MMV) Malaria Box, a collection of 400 chemically diverse compounds with known antimalarial (against asexual stage) activity^32^. This open-access resource has been screened in most gametocytocidal assays to date, and the results are publicly available^23^,^24^,^26^,^27^,^28^,^33^,^34^,^35^,^36^,^37^. As expected, the reported identity and potency of gametocytocidal compounds within this collection are broadly discrepant.

We have chosen 3 mature stage V gametocytocidal assays that are routinely used in our laboratory and that rely on different technologies and/or approaches, namely a luciferase-based assay, a high-content imaging (HCI) assay and a female gamete formation assay (Table 1), to test a selected panel of 39 compounds from the MMV Malaria Box and compare the different assay performances. The compounds were handled as per our standard protocols and dispensed using the same equipment for all assays. To eliminate the influence of assay-unrelated variables as much as possible we carried out the assays using parallel cultures, identically induced and manipulated. The assays were executed under identical conditions of haematocrit and gametocytemia at conditions approximating the Standard Membrane Feeding Assay (SFMA)^38^,^39^, the current gold standard transmission blocking assay.

## Results

**Figure 1:**
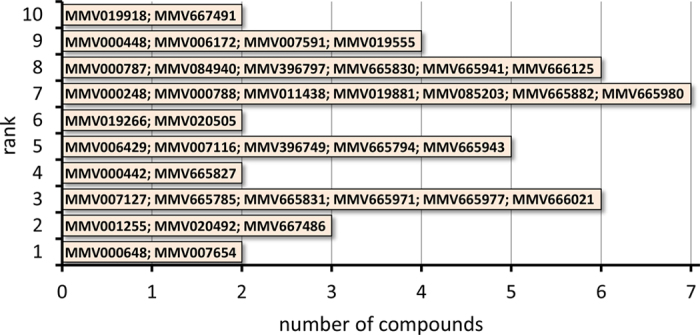
The Gametocytocidal Comparison Set (GCS) compounds. Bars represent the number of compounds selected for each rank (number of published gametocytocidal assays identifying the corresponding compounds as hits).

A panel of 39 antimalarial compounds (in this work referred to as Gametocytocidal Comparison Set, GCS), selected from the MMV Malaria Box^32^ (Fig. 1, see Materials and Methods section for details on the selection procedure), was tested using three established gametocytocidal assays (Table 1). To ensure that all the assays were sufficiently robust, we calculated the Z′ of each plate for each of the assays. The HCI viability assay (GFP-MTR) showed a Z′ range between 0.52–0.62. The luciferase assay (LUC) was more robust with a Z′ range between 0.90 and 0.93. The HCI female gamete formation assay (AO-GMT) had Z′ ranges of 0.74–0.78, 0.76–0.77 and 0.67–0.80 for the 24 h, 48 h and 72 h compound incubation times, respectively. The Z′ values indicate that all the assays ran as expected and were reproducible. A potential bias, a skewed pattern of hit identification, caused in our study by carrying out the different assays in a single laboratory and under the same culturing conditions cannot be excluded a priori. To ensure that no such bias existed, we correlated the GCS compounds ranking utilized to select the GCS set with a similar ranking system obtained by counting the number of assays in the present work that detected each compound as a hit (Supplementary Table S1). A significant correlation was observed at both 5 μM (τb ± SEM = 0.518 ± 0.083; *P* < 0.0001) and 10 μM (τb ± SEM = 0.639 ± 0.077; *P* < 0.0001) suggesting that no major bias was introduced in the hit identification pattern by our specific assays and settings.

### Effect of the screening concentration on inter-assay consistency

**Figure 2:**
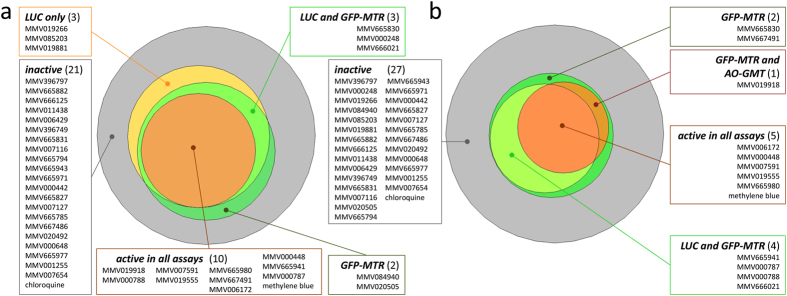
Area-proportional Venn diagrams showing activity distribution of the GCS compounds across assays at 10 μM (**a**) or 5 μM (**b**) after 24 hours incubation and using an activity threshold of 50% inhibition of the relevant normalized assay signals. The number of active compounds is shown in brackets for each assay.

At a 24 h compound incubation time, setting a 50% inhibition threshold at 10 μM and 5 μM concentration resulted in the identification by all assays of 10 and 5 hits, respectively, out of the total 39 GCS compounds (Fig. 2; full inhibition data available in Supplementary Table S2). The control compound, methylene blue, was identified as active by all assays at both concentrations. In contrast, chloroquine, an antimalarial drug known not to possess inhibitory activity against late and mature stage gametocytes, showed less than 23.3% inhibition in all assays at both concentrations.

At a screening concentration of 10 μM, five compounds were identified as assay-specific, i.e. showed ≥50% activity in only one or two of the three assay (Fig. 2a). The LUC assay detected three specific hits (MMV019266, MMV085203 and MMV19881), while the GFP-MTR assay exclusively detected two other compounds (MMV084940 and MMV020505). Additionally, both the GFP-MTR and the LUC assays detected 3 compounds (MMV665830, MMV000248 and MMV666021) which were not detected by the AO-GMT assay.

At 5 μM screening concentration, the GFP-MTR assay detected two compounds, namely MMV667491 and MMV665830 with 51.8% and 56.8% inhibition respectively, which did not show inhibition beyond the set threshold in the other two assays. The LUC and GFP-MTR assays were both able to detect 4 compounds (MMV665914, MMV000787, MMV000788 and MMV666021) that were not picked up by the AO-GMT assay, while one compound (MMV019918) was detected by the AO-GMT and GFP-MTR assay, but not by the LUC assay. Compound MMV665830, which at 10 μM was detected by both the LUC and GFP-MTR assays, was detected by the GFP-MTR assay alone at a lower screening concentration of 5 μM. All compounds that were detected by the gamete assay were also detected by both (10 μM) or at least one (5 μM) of the other two assays (Fig. 2b). Based on the total number of hits, the GFP-MTR assay appeared to be the most sensitive among the three assays, a feature that could be advantageous to detect initial actives from screening. In contrast, the AO-GMT was the least sensitive assay, detecting a lower number of compounds, all of which were shared hits with the other assays. This ‘conservative’ assay was an ideal candidate to subsequently test the effect of compound exposure time.

To better appreciate the specificity of the assay-specific hits for each of the assays and to validate the results, full dose-response data were generated (Supplementary Fig. S1). Only compound MMV666021 appeared to be specific for the LUC and GFP-MTR assays (IC_50_ = 0.67 ± 0.05 μM and 0.99 ± 0.14 μM, respectively), with no detectable effect in the AO-GMT. To understand the discrepancy with the AO-GMT assay, images from this assay and the GFP-MTR were inspected. This revealed that MMV666021 did not alter the morphology of the gametocytes, nor their ability to round-up after xanthurenic acid stimulation, however it suppressed the Mitotracker Red signal, so that the GFP-MTR assay showed a dose-dependent reduction in gametocyte counts (Supplementary Fig. S2). MTR is used in the GFP-MTR assay to detect gametocyte viability and a reduction in MTR fluorescent levels is suggestive of dead or dying parasites, which may or may not lose their elongated shape along with the MTR intensity. The LUC readout also showed a dose-dependent reduction in the luminescence output (i.e. reporter luciferase expression) by MMV666021-treated gametocytes, with similar potency as the GFP-MTR assay. Taken together, these observations suggest that the alteration in MTR and LUC signal reflects an early sign of viability loss in the gametocyte population, which does not affect rounding-up, but may possibly bear consequences for the subsequent fitness of affected gametocytes.

Most other ‘assay-specific’ hits showed some degree of dose-dependent inhibition in all assays, demonstrating a partial selectivity, rather than absolute specificity by the compounds. Indeed, all of these hits were weak inhibitors which exceeded the 50% threshold in one or two assays, but did not reach a complete inhibition plateau in any of the assays.

### Comparison of hit potency with different assay technologies

**Figure 3:**
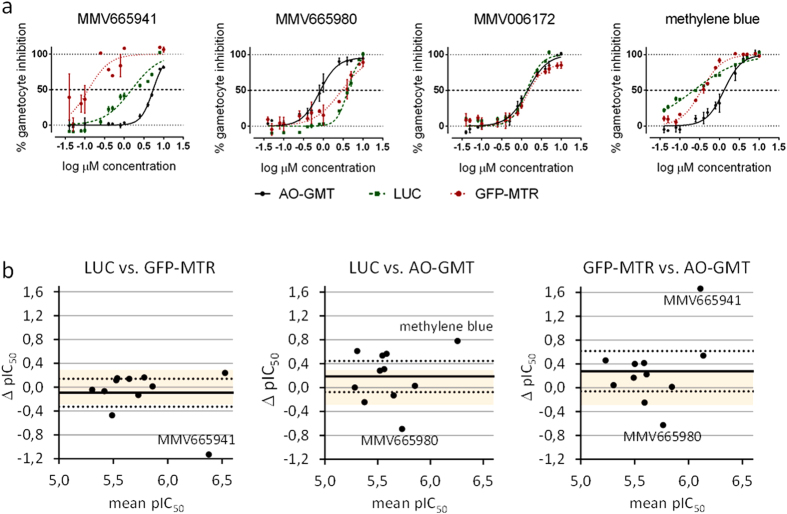
Dose-response curves of 3 representative GCS compounds identified as hits by all assays at 10 μM (Full IC_50_ values in Supplementary Table S2) (**a**). Bland-Altman plots of pairwise comparison of the potencies of the GCS hits determined in different assays (**b**). Continuous and dotted reference lines in (**b**) represent average pIC_50_ bias and its 95% confidence interval limits, while shaded area indicates a pIC_50_ bias range corresponding to a 2-fold IC_50_ ratio.

The potencies of the 10 compounds which demonstrated ≥50% inhibition at 10 μM in all the three assays were compared. Four representative compounds are shown in Fig. 3a and complete dose-response data is available in Supplementary Fig. S3 and Supplementary Table S3.

Generally good agreement was observed in the IC_50_ values obtained from the three assays, with mean ΔpIC_50_ ± SEM of 0.12 ± 0.01 across compounds and assays. The ΔpIC_50_, however, also showed a wide range, from −1.13 to 1.67, i.e. corresponding to IC_50_ shifts of more than one order of magnitude.

Compound MMV006172 showed the most similar inhibition curves and IC_50_ values of all the 10 hits across the three assays. In contrast, MMV665941 showed the highest divergence among assays, especially in the GFP-MTR assay.

Overall, the LUC and GFP-MTR assays showed the best agreement in Bland-Altman plots (Fig. 3b), with the lowest average pIC_50_ bias of −0.09 and the narrowest spread (95%CI = −0.36–0.17). The comparison of the LUC and GFP-MTR assays with the AO-GMT assay showed higher average pIC_50_ biases of 0.19 (95%CI = −0.11–0.48) and 0.28 (95%CI = −0.11–0.66).

As already observed, MMV665941 was the major outlier in the Bland-Altman plot of the GFP-MTR assay in comparison to the other two assays, showing a ΔpIC_50_ of 1.13 vs LUC (corresponding to a 13.5-fold IC_50_ shift; *P* < 0.0001) and 1.67 vs AO-GMT (46.3 fold shift; *P* < 0.0001; Fig. 3a,b). The IC_50_ values for this compound were ∼5.3 μM, ∼1.5 μM, and 0.114 ± 0.026 μM in the AO-GMT, LUC and GFP-MTR assays, respectively (Supplementary Table S3). The visual inspection of the images from this assay and the AO-GMT assay revealed that similarly to MMV666021, MMV665941 also suppressed the MTR signal in the GFP-MTR, leading to the identification of only a fraction of the parasites (Supplementary Fig. S4, compare with the negative control 0.4% DMSO in Supplementary Fig. S6). This is again suggestive of a subtle effect on gametocyte viability that is picked up with higher sensitivity by the GFP-MTR assay. The GFP-MTR dose-response curve for the compound, however, also showed a more erratic point distribution suggestive of additional artefacts or confounding factors in the GFP-MTR assay compared to the other two assays.

MMV665980 was an outlier in the AO-GMT assay compared with the LUC and, less pronouncedly, the GFP-MTR assay, with IC_50_ values of 0.836 ± 0.245 μM, ∼4.1 μM (ΔpIC_50_ = 0.69; 4.9-fold IC_50_ shift; *P* < 0.0001) and ∼3.5 μM (ΔpIC_50_ = 0.63; 4.2-fold shift; *P* < 0.0001), respectively (Supplementary Table S3). Images from the two HCI assays were also inspected and no obvious artefacts were detected (Supplementary Fig. S5). This could be interpreted as the compound having a stronger inhibitory effect on gamete formation than on gametocyte viability. The positive control compound methylene blue was also an outlier between the AO-GMT and the LUC assay and, to a lesser degree, the GFP-MTR assay. The corresponding IC_50_ values were 1.356 ± 0.482 μM, 0.226 ± 0.031 μM (ΔpIC_50_ = −0.78; 6.0-fold IC_50_ shift; *P* < 0.0001), 0.391 ± 0.001 μM (ΔpIC_50_ = −0.54; 3.5-fold shift; *P* < 0.0001) in the AO-GMT, LUC and GFP-MTR assays respectively.

Minor outliers included MMV019918, which was ∼3-fold more active in the GFP-IMG vs the LUC assay (*P* < 0.0001), as well as and MMV000788, which was ∼4-fold more active in the LUC compared to the AO-GMT assay (*P* < 0.0001). MMV000787 also showed differences between the assays, however its potency was modest in all assays, with the lowest IC_50_ value of 2.4 μM in the LUC assay and just 50% inhibition at 10 μM in the AO-GMT assay.

### Effect of incubation time

**Figure 4:**
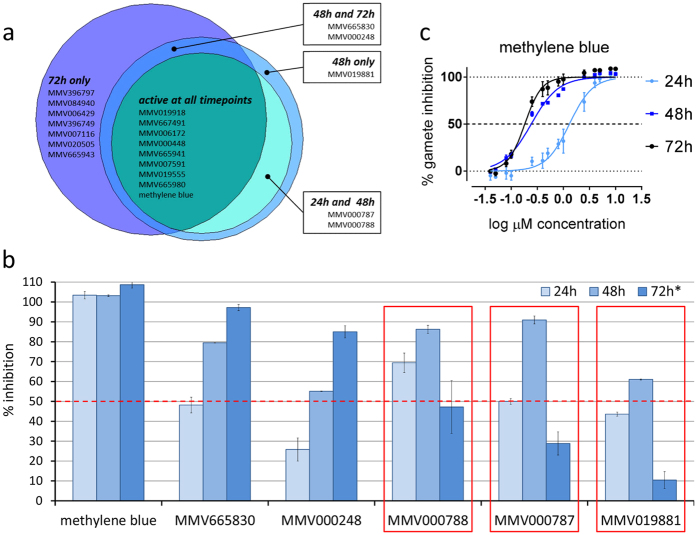
Effect of incubation time on activity of GCS compounds in the AO-GMT assay. Area proportional Venn diagram showing the active compounds identified at three incubation times at 10 μM (24 h, 48 h and 72 H) (**a**). Selected compounds and activity threshold set at 50% inhibition (**b**). Unstable compounds are highlighted in (**b**). Dose response of methylene blue in the AO-GMT assay with different incubation times (24 h, 48 h and 72 h) (**c**). *Indicates an additional freeze/thaw cycle of test compounds.

To investigate the effect of compound incubation time on assay outcomes, the GCS was additionally tested at 48 h and 72 h incubation in the AO-GMT assay. In comparison to the 24 h incubation time, 3 and 7 additional compounds showed activity ≥50% at 10 μM at the 48 h and 72 h time points, respectively (Fig. 4a, Supplementary Table S4). Of the compounds demonstrating activity at 72 h, two were also active at the 48 h incubation. In contrast, compounds MMV000788 and MMV000787 had ≥50% activity after both 24 h and 48 h incubation, but not 72 h, and MMV019881 only had activity above threshold at the 48 h incubation period (Fig. 4b and Supplementary Fig. S8). A 1.9-fold overall increase in detection of actives compared to the 24 h incubation time was observed at 10 μM. The corresponding increase in actives detection at 5 μM was 2.3-fold.

A remarkable increase in potency with longer incubation times was observed for two compounds: MMV007591, from ∼4 μM at 24 h to 0.868 ± 0.020 μM at 72 h (4.8 fold change; *P* < 0.0001) and for the control compound methylene blue (Fig. 4c), from 1.357 ± 0.246 μM at 24 h incubation to 0.242 ± 0.004 μM (5.6 fold change; *P* < 0.0001) and 0.226 ± 0.059 μM (6.0 fold change; *P* < 0.0001) at 48 h and 72 h incubation, respectively, bringing the value closer to the 24 h IC_50_ obtained for this compound with the other two assays (Supplementary Table S5). A moderate increase in potency was observed for MMV019918, from 3.18 ± 0.07 μM at 24 h to 1.00 ± 0.04 μM at 72 h (*P* < 0.0001).

### Identification of compounds with transmission-blocking activity

A standard membrane feeding assay (SMFA) with increased throughput using luciferase-expressing transgenic parasites was recently reported and used to assess a sample of the MMV Malaria Box for reductions in *P. falciparum* transmission to *An. stephensi* mosquitoes^40^. The GCS subset used in this work includes the 18 compounds tested in the SMFA report. To what degree the different assays and parameters tested in this study translate to the identification of transmission-blocking compounds, or in other words, the level of agreement between the outcomes of our gametocyte assays and the SMFA remains to be confirmed. The acknowledged difficulty in comparing data of assays that measure only gametocyte viability from different laboratories is further complicated in this case by the limited overlap in the biology covered by our gametocyte assays (gametocyte viability and gamete formation) and SMFA (from gametocyte viability to oocyst development). For this reason, a quantitative comparison between the data obtained in our work and the published SMFA data was not attempted. As a way to estimate potential for transmission blocking identification, we defined ‘agreement’ as the ability of the compounds to produce a dose-dependent reduction of gametocytes on one hand and of oocysts on the other.

Sixteen compounds were found to be active in SMFA using a washout format, i.e. pre-exposing gametocytes for 24 h and then removing the compounds before the mosquito infection. Of these, only 5 active compounds were identified by us in all three assays after 24 h incubation (Table 2). Interestingly, this number increased to 7 when considering compounds that were identified by at least one of the assays and to 9 when considering compounds active at any incubation time in the AO-GMT assay. Consequently, a significant improvement in the agreement between the HTS assays and the SMFA was observed. The transmission blocking predictivity improved from 39% when considering only compounds agreeing with SMFA in all assays at 24 h to 67% when also including the compounds agreeing with SMFA in any of the assays at 24 h and at any time point over 72 h incubation in the exemplar AO-GMT assay (Cochran’s Q = 26.615; Df = 4; *P* < 0.0001). This corresponded to a level of agreement between HTS and SMFA such that their difference was not any more significant (*P = *0.085 for the Cochran’s test pairwise comparison).

## Discussion

Three different *in vitro* HTS assays interrogating the activity of compounds against *P. falciparum* gametocytes were compared to evaluate the contribution of technology and assay parameters to the assay outcomes. Conditions allowing the direct comparison of the assays were ensured by excluding potential interference associated with variable induction protocols and culturing approaches, compound concentration, incubation length and data analysis. A subset of the MMV Malaria Box compounds, previously reported to possess activity against late stage gametocytes, was used for this comparison. We chose diverse and well validated technologies, namely a luciferase-based assay^36^ (LUC) and two HCI assays differing substantially in approach, one being based on mature gametocyte mitochondrial activity (GFP-MTR)^23^ and the other on female gamete formation (AO-GMT)^27^. In addition, assay conditions that resembled those of the current gold standard, the standard membrane feeding assays (SMFA)^38^,^39^, were employed, specifically the use of mature stage V gametocytes and a short compound incubation time of 24 h. To date, only two reports have attempted to compare the gametocytocidal activity of compounds by different technologies, and were both based on biochemical readouts. One such report by Reader *et al*. used ATP, resazurin, pLDH and luciferase assays against gametocytes at stages IV-V, with a 48 hour incubation^41^. Their results, which were based on % inhibition only and thus did not allow to compare threshold effects or potency, indicated that the compounds tested had a lower activity in the pLDH and ATP assays in comparison to the luciferase and resazurin assays. The second report, by D’Alessandro *et al*., compared recently established luciferase and pLDH assays against early and late stage gametocytes in dose-response at a fixed incubation time of 72 h^25^. This study showed an excellent agreement between the two assays, which measure different biomarkers. In this study, our aim was to gain a deeper understanding of the role that multiple and divergent technologies, including high content imaging, as well as factors such as incubation time, have on the ability to detect hits, compare potency of compounds and predict transmission blocking activity in the mosquito.

Given the very different biological processes probed by our three assays (mitochondrial function, *pfs16* expression and phenotypic changes), it is expected that the assays would not necessarily identify the same compounds in an unbiased screen. The MMV Malaria Box is a collection of compounds selected to represent chemical diversity and antimalarial activity^32^, and is enriched in compounds with gametocytocidal activity^24^,^36^. The GCS subset was further selected to include compounds identified as gametocytocidal by a range of 1 to 10 independent published assays. We found a significant correlation in the frequency the GCS compounds were identified as active between our assays and the published reports, indicating that the information gained from the combination of assays used here can be considered as representative for screening collections enriched with antimalarial compounds. Two screening concentrations of 5 μM and 10 μM, commonly used for gametocytocidal assays, were used and a threshold of 50% inhibition was set to compare hit rates. Differences were observed in the identity and number of hits identified by different assays at both concentrations, however the proportion of ‘assay-specific’ hits did not change substantially between the two screening concentrations. About 40% of the hits (8 out of 18 compounds showing any activity) were picked up by only one or two assays at 10 μM and about 60% (7 out of 12) at 5 μM. This suggests that increasing the screening concentration may not play a major effect in the inter-assay comparability. This is especially true considering that the GCS (and the Malaria Box in general) consists of compounds with rather weak late stage gametocytocidal activity, with IC_50_ values in the low micromolar range, a level close to the screening concentration(s), and therefore expected to show strong threshold effects. When promising candidates with potent activity are present in a library, such as the control compound methylene blue in our study, they are consistently detected by all assays and the difference seen between two high screening concentrations such as 5 and 10 μM are expected to be negligible for successful detection of such compounds.

When examining the potency of these ‘assay-specific’ hits, it appeared evident that the ‘specificity’ was only the effect of the application of an arbitrary cut-off on the weak inhibitors in the set. This was again an effect of the low potency of the experimental compounds, which in many cases showed inhibition around the hit threshold only at the highest screening concentrations. Only one compound, (MMV666021, 5% of the collection) showed complete selectivity for two of the three assays, and any screening concentration above 1 μM would have detected this difference.

The potency of hits that were identified by all assays showed an overall wide ΔpIC_50_ range, from −1.13 to 1.67. However, the small average ΔpIC_50_ value of 0.12 ± 0.01 suggested a scenario of general good agreement, with few outliers.

Overall, each assay detected some compounds with partial or complete selectivity compared to the other assays. Such compounds included MMV666021 (GFP-MTR + LUC), MMV665941 (GFP-MTR) and MMV665980 (AO-GMT). This highlights the usefulness of carrying out screening or hit confirmation activities using multiple, complementary technologies.

Another important criterion for the comparison of gametocytocidal assays is the length of incubation with compounds. In our study, all the compounds were incubated with parasites for 24 h to mimic SFMA conditions. Most published assays, however, involve incubation times of 48–72 h^18^,^19^,^20^,^21^,^22^,^26^,^28^,^29^,^30^. Our three assays were also originally developed and utilized for screening with 48 h^27^ or 72 h^23^,^36^ compound incubation time. To appreciate the effect of incubation time in relation to compound activity we carried out the AO-GMT assay with additional 48 h and 72 h compound exposure times. Incubation time appeared to greatly improve the ability of this less sensitive assay to detect actives, with a 1.9-fold increase in hit numbers at 10 μM, and >2-fold increase at 5 μM. The additional compounds found to be active in this assay at 10 μM and 72 h included the GFP-MTR ‘assay-specific’ compounds MMV084940 and MMV02505. The fact that increasing the incubation time of the least sensitive assay resulted in the detection of actives that have been exclusively detected in the other two assays at 24 h indicates that incubation time might play an equally important role as assay technology in the capability to identify hits, by increasing the sensitivity of the assay.

On the other hand, an increase in potency between 24 h and 72 h incubation (based on a threshold of 5-fold change in the IC_50_, which in our experience is sufficient to rule out intrinsic assay variability) was found only for two compounds, including the control methylene blue. This observation, although limited to a single assay and a small number of compounds, might suggest that while drastically improving the ability to detect actives, a longer incubation time may not cause large deviations in the estimated potency of the hits.

Mature gametocytes are cells in a suspended developmental state, which are sensitive to changes in their environment and ready to respond^42^. If the right cues are provided, such as exposure to xanthurenic acid and a drop in temperature, mature gametocytes rapidly activate and round-up within few minutes^27^. The molecular machinery required for this process is already stored in the cell, awaiting to be triggered. A short incubation time with compounds might not be sufficient for some gametocytocidal actives to disrupt this machinery or its function.Thus, as the main readout of the AO-GMT assay is the activation and rounding-up of the female gametocyte, this assay may fail to identify such actives. On the other hand, the LUC assay is based on the measurement of luciferase activity driven by the expression of the reporter gene *pfs16*^43^. Although transcriptional and translational activities are supposed to be limited in quiescent mature gametocytes^10^, these processes appear to be appropriate indicators of gametocyte viability and more sensitive than rounding-up. The GFP-MTR assay, based on the measurement of MitoTracker Red accumulation in active gametocyte mitochondria, showed that mitochondrial activity closely reflects gametocyte viability and also proved to be a sensitive tool for the detection of gametocytocidal actives at 24 h. While the LUC and GFP-MTR assays were more sensitive than the AO-GMT assay at the 24 h time point, our experience indicates that a prolonged compound incubation time results in the detection of an increased number of actives by these assays, as well. To be able to reach its molecular target and exert its biological effect in malaria parasites, a compound must potentially cross multiple membranes, and may have to accumulate to essential concentrations in relevant cellular compartments. In addition, a delay could exist between the inhibition of the target and the phenotypic changes resulting from the altered downstream processes. A longer incubation time may therefore be required to allow these processes to complete, as previously illustrated using an unrelated assay technology, such as pLDH^19^.

Three compounds that were active at 24 h and 48 h incubation time were no longer active at 72 h (MMV019881, MMV000787 and MMV000788). A likely reason for this apparent loss of activity is due to the handling of the compounds, since the 72 h time point was carried out on a separate occasion and involved one additional freeze-thaw cycle of the compounds DMSO stocks.

This shows that compound handling plays an essential role for the success in identifying and characterizing actives. Precipitation of compounds at each freeze-thaw cycle might result in a lower, inaccurate concentration of compounds in the final assay. This likely accounted for the observed drop in activity at 72 h^44^,^45^. Hence, it is imperative that care is taken in maintaining chemical libraries frozen when transferring them between laboratories, and that screening is carried out on fresh material, perhaps followed by snap-freezing to limit degradation if the same material is to be used for follow-up studies^46^. Potency determination should ideally be carried out using resynthesized hit compounds.

The estimation of a compound’s *in vitro* efficacy and potency are seldom directly translatable to *in vivo* studies. The SMFA is and will remain the gold standard for transmission blocking activity assessment, and great improvement in the throughput of SMFA have been recently achieved^40^,^47^.

*In vitro* HTS gametocytocidal assays, however, have the advantage of allowing the cost-effective screening of large chemical libraries, a capability unlikely to be matched by SMFA, and they will continue to play an important role in gametocytocidal drug discovery. Attempts at improving the predictivity of HTS assays are therefore important to maximize the yield of potential malaria transmission-blocking candidates from screening. Unfortunately, no single *in vitro* HTS assay against either gametocytes or gametes has so far demonstrated a high degree of accuracy in predicting transmission-blocking effects in mosquitoes^40^.

**Figure 5:**
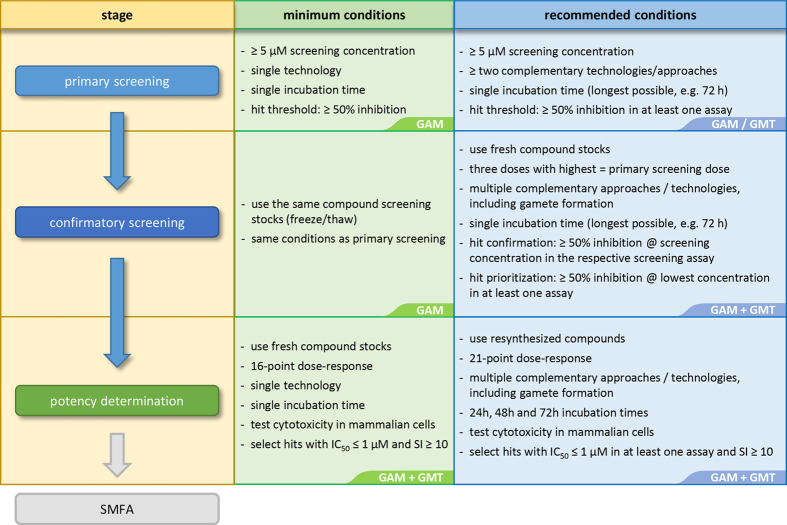
Proposed pipeline for malaria transmission-blocking screening campaigns. While optional for primary screening, the evaluation of compounds in a gamete formation assay is recommended at the confirmation step, and essential at the step of potency determination, before candidates are submitted to SMFA. Cytotoxicity against mammalian cell lines should also be assessed to select candidates based on their selectivity index. GAM = gametocyte viability assay(s); GMT = gamete formation assay(s); SI = selectivity index (mammalian cells IC_50_/gametocytocidal IC_50_); SMFA = standard membrane feeding assay.

Our results suggest that attempting to match the SMFA conditions *in vitro* or relying on a single assay can result in an underestimation of the transmission-blocking potential of the compounds being screened. In fact, utilizing multiple technologies and/or assay approaches in parallel leads to an increase in the number of relevant transmission-blocking compounds found, most likely because of complementarity, rather than redundancy, in the approaches used. In addition, increasing the incubation time with compounds leads to improved detection of relevant, SMFA-active hits. The better agreement between SMFA and HTS with 72 h incubation, as opposed to SMFA-like exposure time of 24 h, shows that each assay has specific requirements for achieving its best predictivity. Hence, SMFA-like conditions should not be taken as an absolute standard when developing HTS assays or designing screening campaigns. Screening compounds at the longest incubation time allowed by the assay, and possibly using multiple assay technologies are both valid approaches towards the identification of relevant transmission blocking hits. A possible gametocytocidal screening cascade and follow-up pipeline is proposed in Fig. 5. Given the low hit rate range of 0.25% − 0.61% reported for gametocytocidal screens of unbiased libraries^26^,^35^,^36^, an increased detection of hits achieved by using prolonged exposure time or multiple technologies is not likely to result in an unpractical number of hits to follow-up, even in the case of some false positives being added along the process.

## Conclusions

There is a definite need for transmission-blocking compounds if the goal of malaria eradication is to be realised. Therefore, assays that can detect gametocytocidal compounds are extremely important. However, current assays have a poor *in vitro* to *in vivo* translation. To improve the agreement between *in vitro* assays and SFMA we recommend to screen compounds using multiple technologies when possible. The two assay approaches should be sufficiently divergent from each other by biology covered, strain and/or technology used. To further increase transmission-blocking predictivity, compounds should be tested at long incubation times. Finally, extreme care needs to be taken in ensuring the integrity of compound stock solutions by good handling and storage practices.

## Methods

### Experimental compounds and assay design

A panel of 39 antimalarial compounds (in this work referred to as Gametocytocidal Comparison Set, GCS) was selected from the publicly available Medicines for Malaria Venture (MMV) Malaria Box^32^. To select the candidates all of the MMV Malaria Box hits that had been identified from gametocytocidal screening in at least one of the 10 late and mature stage gametocyte assays published to date^19^,^23^,^26^,^27^,^28^,^33^,^34^,^35^,^36^,^37^ were collated, and ranked based on the number of times they were identified as hits. For the ranking no attempt to normalize for inherent differences of each assay, such as compound exposure time, concentration tested, etc. was made, instead we relied on the hit definition parameters set by the respective assay developers (Table 3). The ranking yielded a broad range of hitting frequency, from 1 to 10, and 2 to 7 representative compounds from each rank were selected (Fig. 1).

The MMV Malaria Box compounds were received in 96-wells plates as 10 mM stock solutions in 100% DMSO. Selected compounds were serially diluted to a 14-point intermediate concentration range of 400 nM–1000 μM in 4% DMSO, in 384 wells clear V-bottom polypropylene storage plates (Axygen). Five μl of the diluted stock at each concentration were transferred from the storage plates to the relevant assay plates using a Minitrak (PerkinElmer) liquid handler, to a final DMSO concentration of 0.4% v/v and a compound dose-response range of 40 nM–10 μM.

### Parasite culturing and gametocyte induction

Two different *Plasmodium falciparum* lines were used, namely 3D7A for the female gamete formation assay and NF54^Pfs16^ (a reporter gene line which expresses a GFP-luciferase fusion under the gametocyte-specific promoter Pfs16^43^) for both the luciferase and the high content imaging assays.

Asexual parasites were cultured as previously described^48^, with modifications. Briefly, parasites were cultured in human 0^+^ RBCs at 5% haematocrit (Hct) and less than 2% parasitemia in RPMI 1640 medium supplemented with 25 mM HEPES (Sigma), 50 μg/ml hypoxanthine, 5% AB human serum (Sigma) and 2.5 mg/ml Albumax II (Gibco). All cultures were incubated at standard conditions, consisting of 37 °C in gas mixture of 5% O_2_, 5% CO_2_ and 90% N_2_.

We utilized our established protocol for gametocytogenesis induction^49^, with modifications consisting in the maintenance of the cultures at 15% gametocytemia and 1% haematocrit (hct) post MACs column isolation on day 8 of gametocytogenesis and daily media exchange until the parasites were used for assays on day 12.

### Gametocytocidal assays

The compounds were tested in two technical replicates and two independent biological replicates for each of the three gametocytocidal assays (Table 2). All assays were set up simultaneously on parallel gametocyte cultures of the relevant strain.

### Luciferase assay (LUC)

The LUC assay is based on the measurement of the bioluminescent activity of luciferase-expressing NF54^Pfs16^ gametocytes. The assay was carried out as previously described^36^, with modifications. Briefly, mature stage V gametocytes on day 12 of gametocytogenesis were seeded in 384 wells white luminescence plates (Culturplate, PerkinElmer) at 0.1% hct and 10% gametocytemia in 45 μl and incubated with compounds for 24 h. In each assay plate, 7 wells containing the gametocytocidal reference compound methylene blue at 10 μM and 7 wells treated only with the solvent 0.4% DMSO were used as in-plate positive and negative controls, respectively. At the end of the incubation, 25 μl medium were aspirated simultaneously from each well and replaced with 15 μl of the homogeneous luciferase reporter gene assay system Steadylite plus (PerkinElmer) without disturbing the settled red blood cells (RBCs), as per our standard method^24^,^36^. Luminescence was measured after 1 hr incubation at room temperature using a MicroBeta Trilux (PerkinElmer) multidetector luminometer.

### High-Content imaging gametocytocidal assay (GFP-MTR)

The imaging assay is based on the detection of elongated gametocytes using the dual staining given by the endogenous GFP expression of NF54^Pfs16^ gametocytes and the mitochondrial stain MitoTracker Red. The assay was performed as previously described^23^, with the following modifications. Mature stage V gametocytes on day 12 of gametocytogenesis were used. Parasites were seeded at 0.1% hct and 10% parasitemia in 45 μl in 384 wells black clear bottom, PDL-coated CellCarrier imaging plates (PerkinElmer) and incubated with compounds for 24 h at standard conditions before the addition of the detection reagent. Seven wells containing the gametocytocidal reference compound methylene blue at 10 μM and 7 wells treated only with the solvent 0.4% DMSO were used as in-plate positive and negative controls, respectively. After the incubation, 5 μl of 0.07 μg/ml MitoTracker Red CMH2XRos (MTR; Invitrogen, Australia) in phosphate buffered saline (PBS) were added to the plates, and these were incubated for 12 additional hours under standard conditions. Plates were then brought to room temperature for at least one hour before being measured on the Opera QEHS Confocal Imaging System (PerkinElmer). Images were taken for each well at 3 μm from the bottom of the imaging plate using a 20X water immersion objective. GFP intensity was measured at an exposure time of 400 msec (488 nm), then MTR signal was measured at 532 nm for 600 msec. An Acapella-based script was optimized to select objects with a MTR fluorescent signal above an assay-optimized cut-off, to ascertain mitochondrial activity as a proxy for parasite viability, and an elongated GFP object shape, as per our standard method^23^.

### High-Content Imaging female gamete formation assay (AO-GMT)

This assays utilizes the fluorescent dye acridine orange (AO) to stain day 12, mature stage V gametocytes after activation with the gamete formation inducer xanthurenic acid (XA)^50^, to assess compound activity on both gametocyte viability and on the process of female gamete formation. The assay was carried out as previously described^27^. Briefly, mature stage V gametocytes were seeded on day 12 of gametocytogenesis at 0.1% hct and 10% parasitemia in 45 μl into 384 wells black, clear bottom Viewplate (PerminElmer) imaging plates, and incubated with compounds at standard conditions for 24, 48 or 72 hours (the latter was performed on a different date and involved one additional freeze/thaw cycle of the compound stocks). Seven wells were treated with methylene blue at 10 μM (full kill control) and 7 wells were treated with the PfPKG inhibitor compound-2, which completely blocks the gametocyte rounding-up process^51^,^52^ at 5 μM (no-activation control), to be used as in-plate positive controls. Seven wells treated with 0.4% DMSO were used as in-plate negative controls. At the end of the incubation, plates were brought to room temperature and the medium was replaced with XA- and AO-containing RPMI to a final in-well concentrations of 40 μM XA and 60 nM AO. Images of stained parasites (gametocytes + gametes) were taken after 2.5 hours light-protected incubation at 22.7 ± 0.3 °C, using the Opera system at 488 nm excitation and 520/35 nm emission, with a 280–400 msec exposure time. A custom Columbus v.2.5 (PerkinElmer) script based on the spot detection algorithm was used to detect and count AO-positive fluorescent spots with intensity beyond an assay-optimized threshold, and to discriminate between elongated (non-activated gametocytes) and round-shaped cells (female gametes).

### Data analysis

Normalized inhibition data were generated by applying the formula:


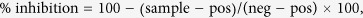


where sample is the raw value obtained from the readout of any well treated with an experimental compound, pos is the mean readout of all the positive control-treated wells from the same plate and neg is the mean readout of all the negative control-treated wells from the same plate.

The performance and reproducibility of the assays were monitored by measuring the %CV and Z′ of the assays^53^, based on the negative and positive controls in each individual plate.

Normalized % inhibitions were plotted against log-transformed μM concentration of each compound and IC_50_ values were calculated using a 4 parameter non-linear regression analysis in GraphPad Prism v. 5.0, using the constraints bottom = 0 and top ≤ 100.

The correlation between the rank assigned to the GCS compounds and the hitting frequency of the compounds in our assays was assessed using a Kendall’s Tau-b algorithm in SPSS v.23 (IBM).

The comparison of the agreement between compounds activity at multiple HTS assay conditions and transmission blocking activity in SMFA was evaluated using the non-parametric Cochran’s Q test for related samples with pairwise comparisons, in SPSS v.23 (IBM).

## Additional Information

**How to cite this article:** Lucantoni, L. *et al*. The need to compare: assessing the level of agreement of three High-Throughput assays against *Plasmodium falciparum* mature gametocytes. *Sci. Rep.* **7**, 45992; doi: 10.1038/srep45992 (2017).

**Publisher's note:** Springer Nature remains neutral with regard to jurisdictional claims in published maps and institutional affiliations.

## Supplementary Material

Supplementary Information

## Figures and Tables

**Table 1:** Summary of the three gametocytocidal assays utilized to compare the activity of the GCS compounds.

Assay	*P. falciparum* line	Gametocyte stage	Incubation time	Additional incubation
luciferase (LUC)^36^	NF54^Pfs16^	mature V	24 h	1 h
High-content imaging gametocyte (GFP-MTR)^23^	NF54^Pfs16^	mature V	24 h	12 h
High-content imaging female gamete (AO-GMT)^27^	3D7A	mature V	24 h, 48 h, 72 h*	2.5 h

^*^The 72 h AO-GMT involved an additional freeze-thaw cycle for the compounds.

**Table 2:** Comparison of the agreement between multiple HTS assay technologies/extended compound incubation time and transmission-blocking reduction in SMFA.

compound	all assays (24 h)	at least one assay (24 h)	longer incubation time (72 h)	overall	SMFA (washout)* IC_50_ (μM)
MMV000248	×	✓	✓	✓	1.64
MMV000442	×	×	×	×	0.59
MMV000448	✓	✓	✓	✓	1.18
MMV007116	×	×	✓	✓	0.11
MMV011438	×	×	×	×	8.58
MMV019266	×	×	×	×	1.29
MMV019881	×	×	✓	✓	1.17
MMV019918	✓	✓	✓	✓	0.07
MMV020492	✓	✓	✓	✓	not converged
MMV396797	×	×	✓	✓	3.62
MMV665827	×	×	×	×	0.1
MMV665882	✓	✓	✓	✓	not converged
MMV665941	✓	✓	✓	✓	0.04
MMV665971	×	×	×	×	3.22
MMV665980	✓	✓	✓	✓	1.76
MMV666021	×	✓	×	✓	1.25
MMV666125	×	×	×	×	1.4
MMV667491	✓	✓	✓	✓	0.06
proportion	0.39^a^	0.50^a^	0.61^a^	0.67^ab^	1.0^b^

Ticks and crosses represent agreement and disagreement with SMFA data, respectively. Agreement is defined as a valid IC_50_ being obtained with HTS assays for SMFA-active compounds, and inactivity for SMFA-inactive compounds. Proportion values represent the number of compounds agreeing with SMFA outcomes at each condition over the total number of compounds. Different letters indicate statistically significant differences between the groups (non-parametric Cochran’s Q test pairwise comparisons at the 0.05 significance level).

^*^SMFA data obtained from Vos *et al*.^40^

**Table 3:** Summary of published whole-cell gametocytocidal assays reporting screening of the MMV Malaria Box.

Reference	Stage	Incubation time (hr)	Additional incubation (hr)	screening concentration (μM)	hit threshold	readout	reagent/technology
Bowman *et al*.^33^	V	72	24	5	85% inhibition	viability	AlamarBlue/fluorescence
Duffy *et al*.^23^	IV-V	72	12	5	50% inhibition	viability	GFP, MitoTracker Red/imaging
Lucantoni *et al*.^36^	IV-V	72	0	5	50% inhibition	viability	luciferase/luminescence
D’Alessandro *et al*.^19^,^37^	IV-V	72	72	3.7	50% inhibition	viability	pLDH/absorbance
Plouffe *et al*.^26^	V	72	∼ 1	12.5	70% inhibition	viability	MitoTracker Red/imaging
Sanders *et al*.^34^	V	48	2.5	10	50% inhibition	viability	SYBR Green I/fluorescence
Sun *et al*.^35^	IV-V	72	24	(dose-response, 46 μM top)	(curve class 1.1, 1.2, 2.1)	viability	AlamarBlue/fluorescence
Lucantoni *et al*.^27^	V	48	3	5	50% inhibition	viability + female gamete formation	Acridine Orange/imaging
Ruecker *et al*.^28^	V	24	0	1	50% inhibition	male gamete formation	time lapse live imaging
Ruecker *et al*.^28^	V	24	24	1	50% inhibition	female gamete formation	Cy3-labeled anti-Pfs25/imaging
