# Supplementary material for: The need to compare: assessing the level of agreement of three high-throughput assays against Plasmodium falciparum mature gametocytes
Source: Sci Rep. 2017 Apr 5;7:45992. doi: 10.1038/srep45992 (PMC5380998; doi:10.1038/srep45992)
Supplement: Supplementary Information [file srep45992-s1.pdf]

The need to compare: assessing the level of agreement of three high-throughput assays against *Plasmodium falciparum* mature gametocytes.

Leonardo Lucantoni[1,^], Sasdekumar Loganathan[1,^] and Vicky M Avery[1,*]

[1]Discovery Biology, Griffith Institute for Drug Discovery, Griffith University, 4111 Nathan, Queensland, Australia

^These authors contributed equally to this work

*Corresponding author V.M.A. (email: v.avery@griffith.edu.au)

Supplementary table S1. Comparison of the hit frequency of the GCS compounds in this study at 24h incubation with the literature data (10 different HTS gametocytocidal assays, see main text for references).

compound name	GCS rank	assay rank	
		5 µM	10 µM
MMV665830	10	2	3
MMV019918	10	1	3
MMV006172	9	3	3
MMV667491	9	3	3
MMV665941	9	3	3
MMV007591	9	3	3
MMV000788	8	2	2
MMV019555	8	0	1
MMV000787	8	0	1
MMV000448	8	1	2
MMV665980	8	2	3
MMV000248	8	0	0
MMV084940	7	0	2
MMV085203	7	2	2
MMV396797	7	0	0
MMV019266	7	0	1
MMV019881	7	0	0
MMV665882	7	0	0
MMV665831	7	3	3
MMV666125	6	0	0
MMV006429	6	0	1
MMV011438	5	0	1
MMV396749	5	0	1
MMV666021	5	0	1
MMV007116	5	0	0
MMV020505	5	0	1
MMV665794	4	0	0
MMV665943	4	0	0
MMV665827	3	0	0
MMV665785	3	0	0
MMV665971	3	0	0
MMV000442	3	0	0
MMV007127	3	0	0
MMV667486	3	2	0
MMV665977	2	0	0
MMV020492	2	0	0
MMV000648	2	0	0
MMV007654	1	0	0
MMV001255	1	0	0
methylene blue	control	3	3

Values represent the number of assays that identified each compound as a hit at the relevant screening concentration.

Supplementary table S2. Percent inhibition values obtained with the three assays at two doses (10 µM and 5 µM) and 24h incubation time.

Compound	LUC				GFP-MTR				AO-GMT			
	10 µM	± SEM	5 µM	± SEM	10 µM	± SEM	5 µM	± SEM	10 µM	± SEM	5 µM	± SEM
MMV665830	65.2	4.5	28.7	0.1	87.6	6.8	56.8	11.2	48.2	4.0	18.5	3.1
MMV019918	77.3	4.5	43.0	3.9	109.4	0.5	100.5	0.5	90.7	0.7	66.6	2.6
MMV006172	115.0	3.9	103.3	2.0	85.3	4.3	80.0	5.4	101.3	2.0	84.9	5.8
MMV667491	115.4	1.3	34.7	1.8	106.9	0.9	51.8	6.1	98.7	0.4	36.9	2.5
MMV665941	111.9	0.4	71.4	1.9	106.6	3.5	112.5	0.0	81.5	0.0	42.7	2.3
MMV007591	114.5	3.6	83.7	1.8	106.9	2.4	104.1	2.1	82.4	0.1	51.4	3.4
MMV000788	116.9	4.2	116.5	4.0	98.8	0.5	94.8	2.3	69.4	4.9	40.0	4.7
MMV019555	114.7	4.2	98.3	3.5	101.4	4.5	81.0	2.9	91.2	0.2	63.8	2.2
MMV000787	119.0	3.0	100.3	1.9	95.4	4.6	70.7	0.8	50.0	1.5	19.4	0.1
MMV000448	103.8	5.2	96.3	4.9	77.4	7.4	61.7	3.3	74.1	5.8	57.0	10.5
MMV665980	100.7	2.5	60.0	4.0	89.1	6.1	71.5	5.0	94.7	2.1	91.2	1.4
MMV000248	60.8	3.3	35.2	2.1	82.2	1.6	47.8	1.3	25.8	5.8	7.4	0.6
MMV084940	32.8	2.7	23.9	1.3	58.0	4.4	28.4	4.6	9.5	2.8	4.1	4.0
MMV085203	55.7	0.6	45.1	0.3	25.1	8.2	27.5	6.4	3.0	0.3	0.7	5.5
MMV396797	26.8	6.9	9.9	2.2	21.5	9.0	15.4	0.5	11.8	4.0	3.5	3.9
MMV019266	49.6	18.6	-6.9	2.6	17.1	8.7	1.2	5.4	6.8	0.3	-1.4	1.9
MMV019881	52.1	0.6	36.4	2.7	46.5	0.3	39.7	6.0	43.6	1.0	32.2	0.7
MMV665882	18.1	6.2	17.5	7.2	29.4	6.4	27.4	4.4	-4.7	5.9	2.2	0.3
MMV665831	10.8	1.7	10.8	0.4	20.7	2.8	27.2	3.2	0.4	3.1	-3.9	3.1
MMV666125	4.1	1.8	0.0	1.3	9.5	0.1	15.3	0.9	-2.0	2.0	0.2	2.0
MMV006429	5.3	1.4	-2.5	2.3	13.8	6.1	15.1	9.4	33.9	4.4	13.5	8.6
MMV011438	-1.0	2.6	-1.9	0.6	41.1	3.0	8.5	15.3	3.5	0.4	-7.4	2.1
MMV396749	-3.6	0.8	-2.6	0.8	15.6	8.4	6.6	11.6	7.0	3.3	4.4	5.3
MMV666021	113.5	3.4	107.1	3.4	113.9	1.4	113.1	0.7	10.0	11.5	11.1	6.7
MMV007116	46.7	3.5	39.2	3.1	39.0	3.0	47.5	2.1	4.5	1.8	11.5	5.5
MMV020505	33.5	1.4	19.3	1.9	52.9	9.5	24.7	6.0	13.0	6.0	1.4	4.7

Supplementary table S2 (cont'd)

Compound	LUC				GFP-MTR				AO-GMT			
	10 µM	± SEM	5 µM	± SEM	10 µM	± SEM	5 µM	± SEM	10 µM	± SEM	5 µM	± SEM
MMV665794	7.0	1.0	1.2	0.0	22.7	4.0	18.4	8.3	-1.6	4.6	-8.6	0.2
MMV665943	43.6	3.6	6.3	1.2	26.0	2.7	12.3	7.1	17.2	2.8	3.5	0.6
MMV665827	44.5	3.8	43.1	4.4	45.5	7.4	42.4	2.7	-0.4	5.5	4.0	8.8
MMV665785	3.9	1.5	6.4	1.2	18.9	4.2	21.1	9.0	-4.6	0.1	-5.6	5.1
MMV665971	33.6	0.8	21.6	0.9	46.5	10.4	20.1	2.1	-4.5	1.6	0.5	4.2
MMV000442	4.9	3.4	10.8	2.4	18.1	4.0	13.6	3.7	-4.7	0.0	-0.6	2.7
MMV007127	30.8	1.8	28.9	0.1	22.1	2.4	11.7	3.1	-3.6	2.2	-7.6	0.7
MMV667486	3.5	2.0	14.4	1.6	0.0	3.1	8.5	2.8	-13.0	2.9	-3.8	3.2
MMV665977	44.1	0.3	40.6	0.4	41.6	9.5	44.2	7.0	15.5	6.4	9.9	7.0
MMV020492	1.6	1.9	0.3	1.4	-2.3	4.7	14.2	7.7	-0.6	3.1	1.6	3.2
MMV000648	-4.3	1.4	-4.6	2.3	8.0	1.2	11.5	10.2	11.2	2.9	-1.1	4.4
MMV007654	-38.5	1.0	-17.3	0.3	20.1	1.5	14.4	3.1	-13.4	6.4	-7.3	4.0
MMV001255	12.8	1.7	11.5	0.2	12.8	7.8	7.9	4.4	1.6	0.6	-10.6	2.7
methylene blue	103.1	0.7	95.0	0.3	98.0	0.1	100.6	0.0	103.4	1.9	98.2	3.3
chloroquine	13.4	0.8	8.2	0.7	11.6	7.3	22.4	21.1	23.3	0.0	2.6	7.0

Highlighted values indicate inhibition ≥ 50% (hit threshold).

Supplementary Figure S1

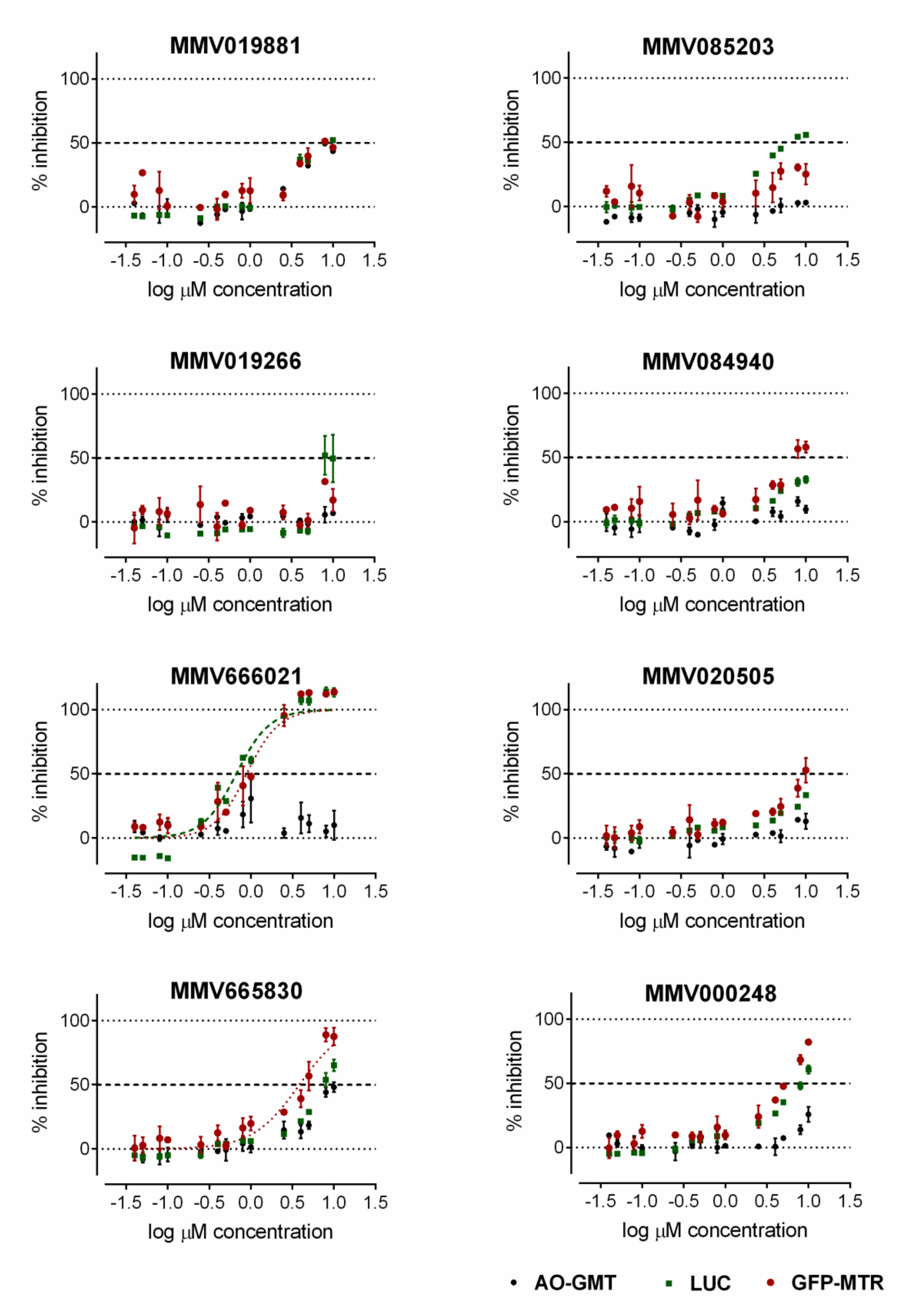

Supplementary Figure S1. Dose-response plots of the assay-specific hits identified using 10 µM concentration and ≥ 50% inhibition as the hit threshold.

Supplementary Figure S2

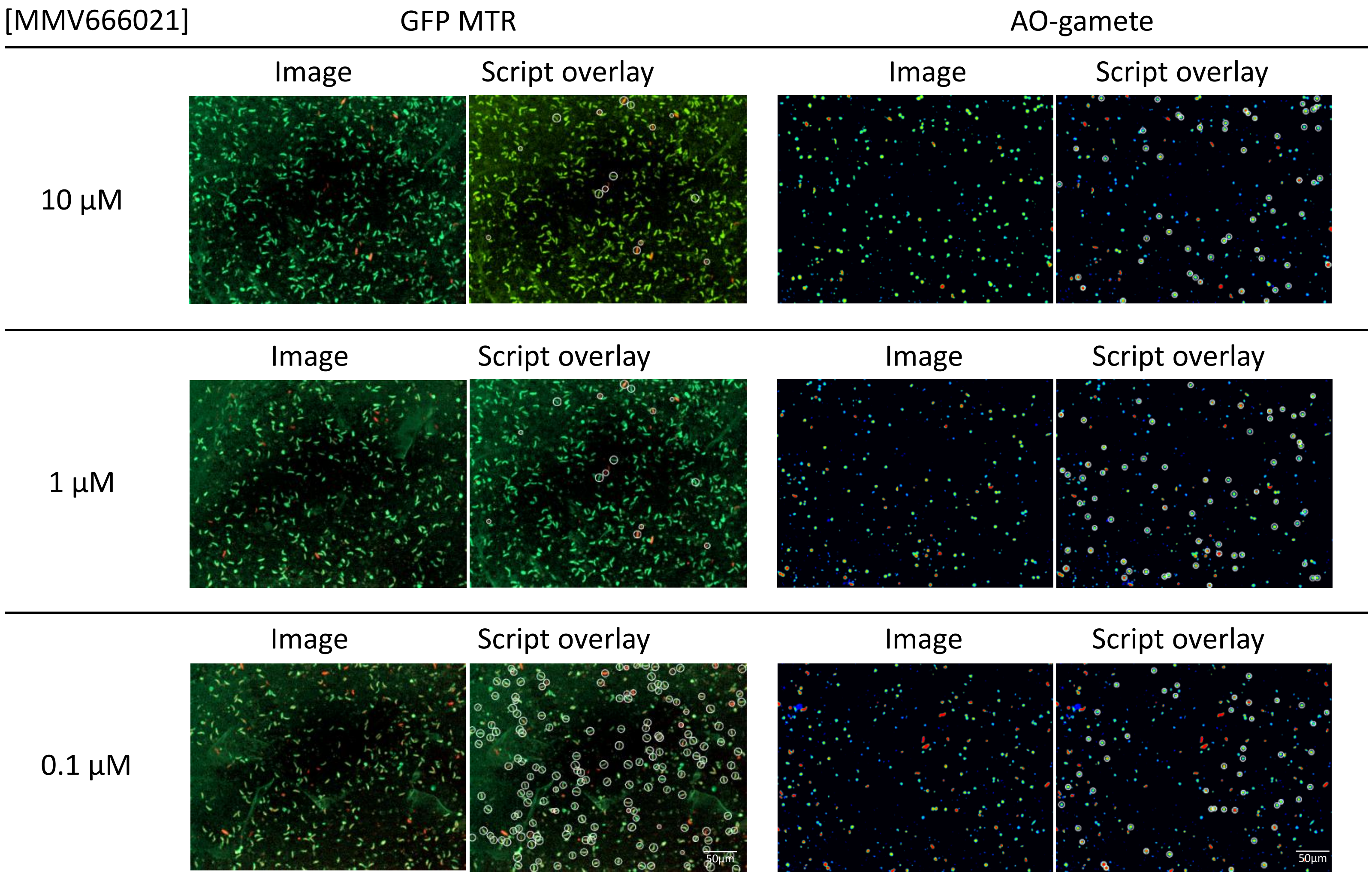

Supplementary Figure S2. Images and image script overlay for GFP-MTR assay and AO-GMT assay for compound MMV665941 at three doses. Parasites detected by the automated image analysis software script are shown as white circles in script overlay images.

Supplementary Figure S3

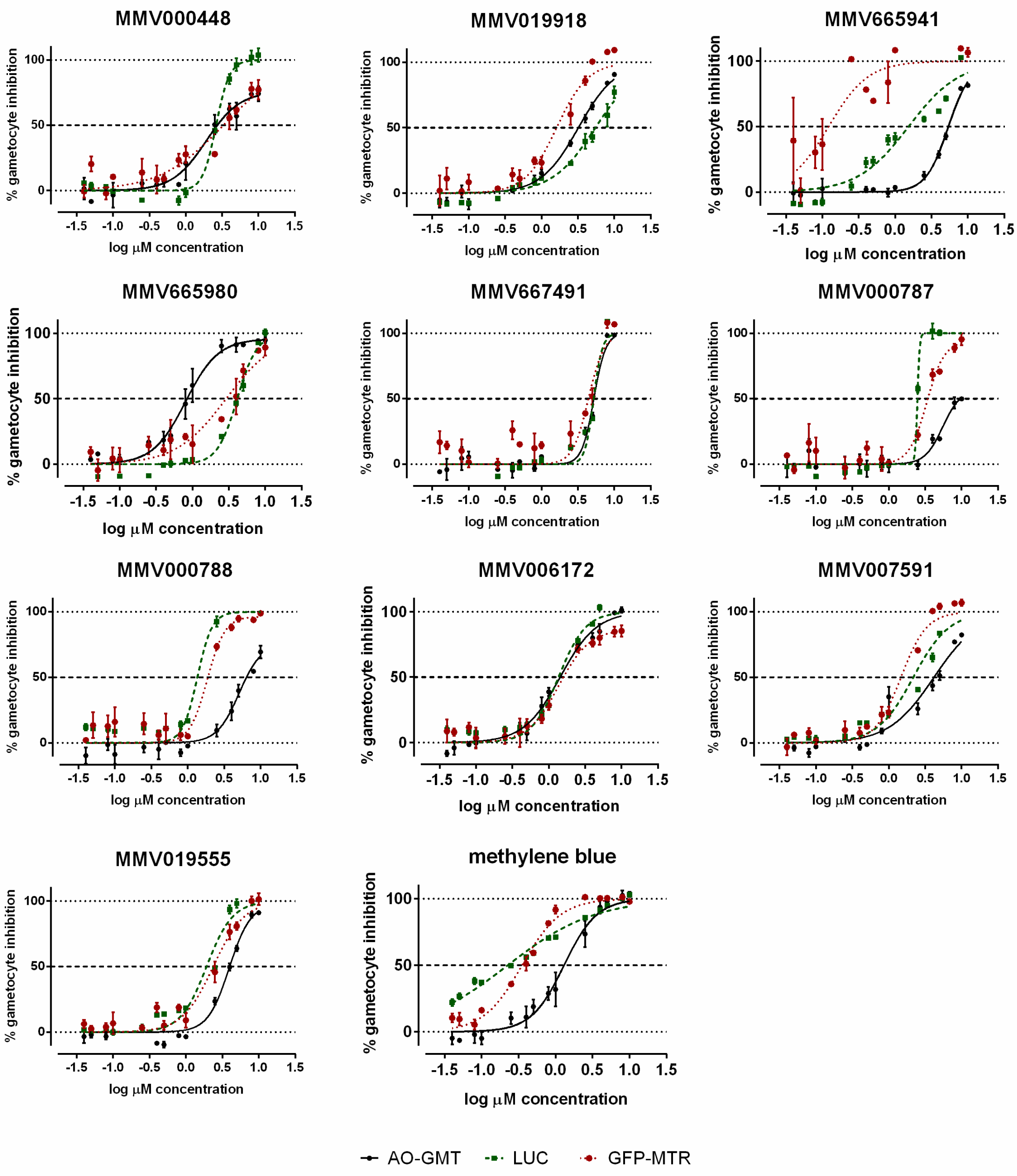

Supplementary Figure S3. Dose-response curves of the 10 GCS compounds identified as hits by all assays using 10 µM concentration and ≥ 50% inhibition as the hit threshold.

Supplementary Table S3 - Potency of the 10 GCS compounds identified as hits (at 10 μM) by all assays at 24h incubation.

Compound	AO-GMT		LUC		GFP-MTR	
	**IC_{50} (nM)**	**± SEM**	**IC_{50} (nM)**	**± SEM**	**IC_{50} (nM)**	**± SEM**
MMV000448	~1921		2602.1	51.1	~3402	
MMV019918	~3177		~5589		1886.6	159.0
MMV665941	~5279		1540.2	246.8	114.1	26.4
MMV665980	836.3	244.6	~4129		~3539	
MMV667491	~5224		~5199		~4712	
MMV000787	50% inhibition @ 10 μM		2445.0	24.8	~3466	
MMV000788	~4991		1362.1	63.8	1982.1	59.8
MMV006172	1451.3	321.8	1360.3	109.2	1400.6	103.2
MMV007591	~4172		~2174		1603.1	63.1
MMV019555	3924.7	140.3	1926.4	31.1	2656.8	427.1
methylene blue	1356.7	482.4	226.1	31.2	391.5	0.9

An estimation of the IC_{50} value is given for curves that did not reach an inhibition plateau.

Supplementary Figure S4

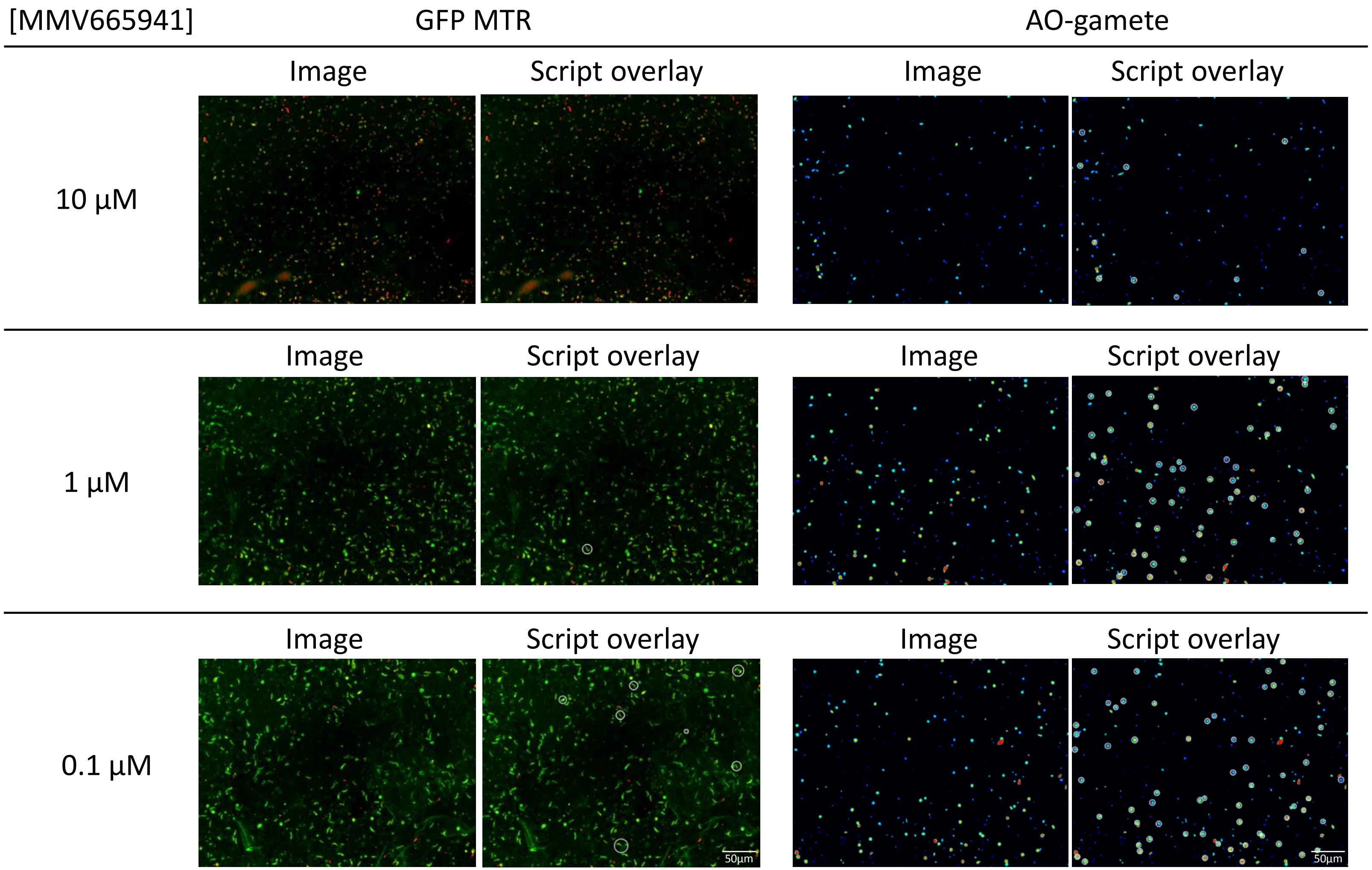

Supplementary Figure S4. Images and image script overlay for GFP-MTR assay and AO-GMT assay for compound MMV665941 at three doses. Parasites detected by the automated image analysis software script are shown as white circles in script overlay images.

Supplementary Figure S5

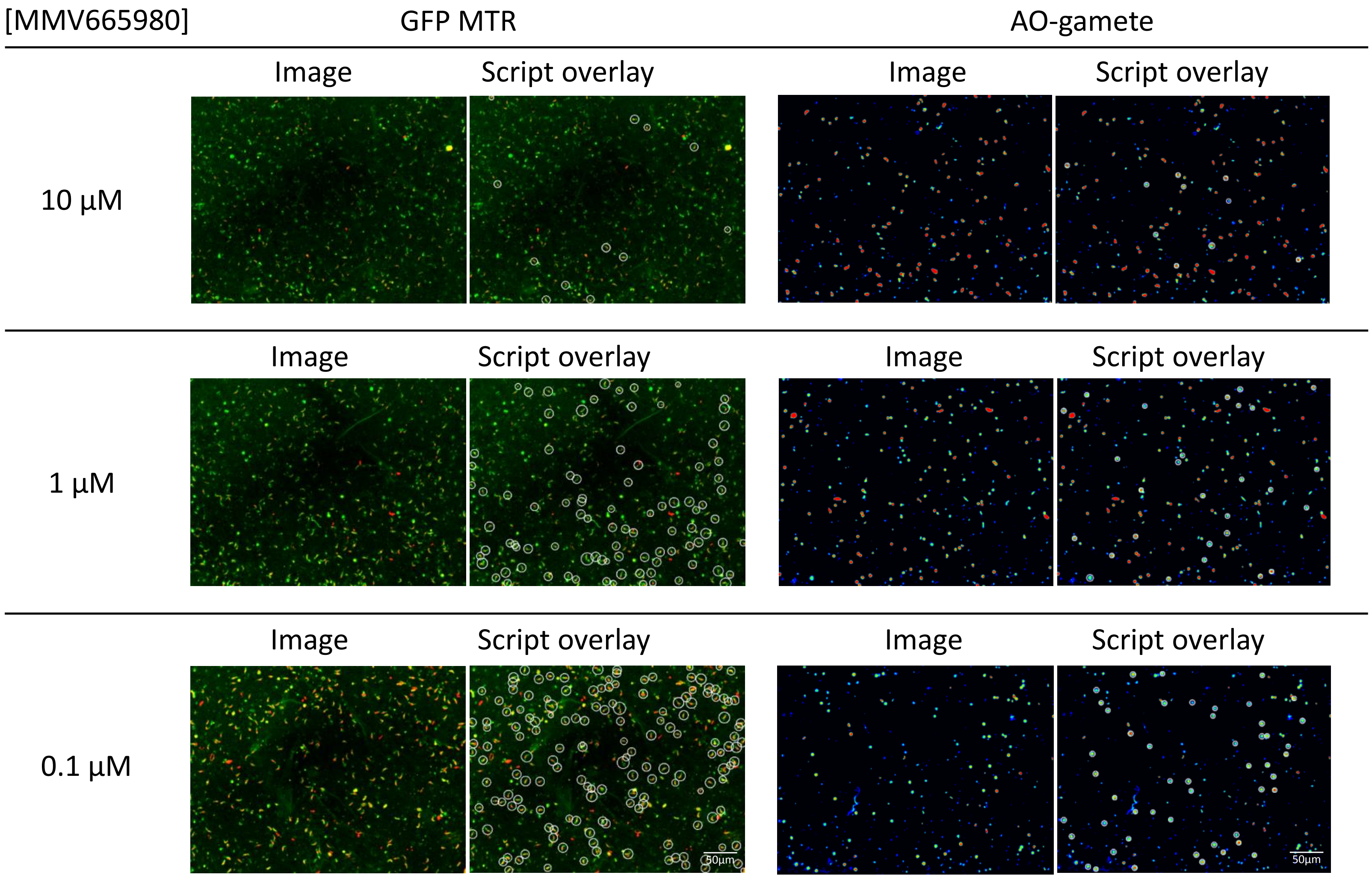

Supplementary Figure S5. Images and image script overlay for GFP-MTR assay and AO-GMT assay for compound MMV665980 at three doses. Parasites detected by the automated image analysis software script are shown as white circles in script overlay images.

Supplementary Figure S6

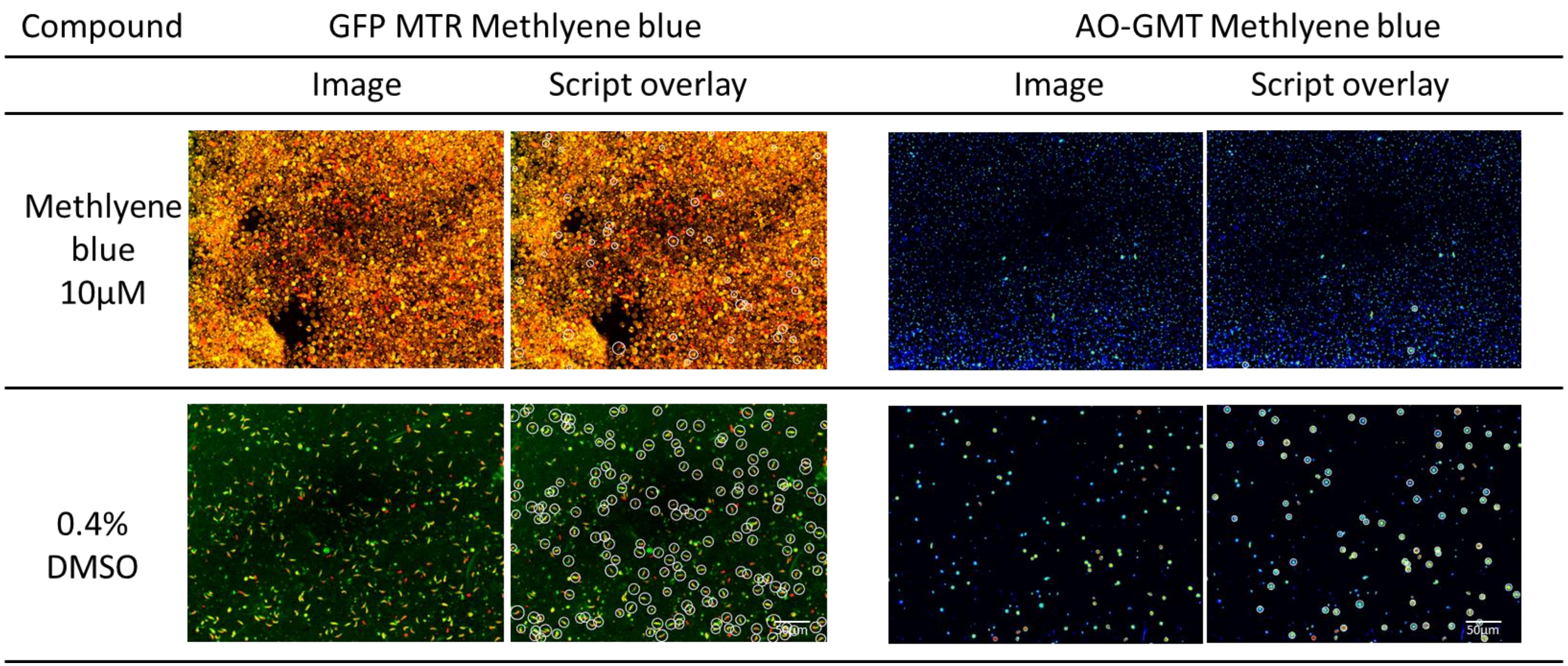

Supplementary Figure S6. Images and image script overlay for GFP-MTR assay and AO-GMT assay for Methlyene blue and DMSO. Parasite are detected by the automated image analysis software script (white circle in script overlay column).

Supplementary table S4. Percent inhibition values of the AO-GMT assay at two doses (10µM and 5 µM) and 48 or 72 hours incubation.

compound	48 h incubation				72 h incubation*			
	10 µM	± SEM	5 µM	± SEM	10 µM	± SEM	5 µM	± SEM
MMV665830	79.5	0.1	31.0	2.1	97.2	1.5	69.0	3.0
MMV019918	99.7	0.5	92.4	0.2	102.8	3.2	98.8	0.7
MMV006172	102.8	0.3	99.6	0.1	105.5	1.0	101.1	1.0
MMV667491	102.0	0.2	55.6	0.5	101.8	1.8	93.5	0.9
MMV665941	90.5	1.1	72.3	2.6	94.5	1.0	81.1	1.9
MMV007591	97.3	0.0	91.4	1.8	98.7	2.0	99.7	1.6
MMV000788	86.2	2.0	83.9	1.1	47.2	13.2	17.8	13.0
MMV019555	100.4	0.6	83.6	1.9	100.7	0.3	93.1	1.0
MMV000787	91.0	2.0	61.7	3.5	28.8	5.8	5.9	4.1
MMV000448	98.5	1.7	82.8	4.5	108.2	1.8	103.5	0.7
MMV665980	97.9	0.3	99.1	0.5	99.0	0.6	98.5	0.1
MMV000248	55.1	0.1	15.5	0.8	85.0	3.1	41.8	0.8
MMV084940	31.8	5.5	12.4	7.2	73.8	1.0	40.1	5.8
MMV085203	40.5	3.7	10.9	9.4	25.5	14.4	3.9	3.1
MMV396797	14.3	3.7	6.0	2.2	93.9	2.9	42.4	7.0
MMV019266	10.4	2.1	6.4	2.1	21.3	10.3	5.6	11.3
MMV019881	61.0	0.3	52.6	0.1	10.4	4.2	0.0	2.5
MMV665882	5.0	1.1	11.1	3.1	-8.1	6.8	-4.5	2.4
MMV665831	5.0	1.4	8.7	3.2	18.0	6.9	15.7	3.5
MMV666125	-4.8	0.4	-5.9	1.0	17.5	3.3	0.6	1.8
MMV006429	29.2	2.6	13.8	1.4	71.8	1.2	63.9	4.7
MMV011438	7.2	3.1	-2.7	4.2	8.8	1.0	-7.2	9.4
MMV396749	-1.1	7.8	3.1	6.9	80.2	1.3	71.8	3.7
MMV666021	11.2	8.6	8.3	8.2	26.6	2.8	0.4	12.5
MMV007116	6.2	7.9	17.8	6.8	64.0	6.7	60.0	3.8
MMV020505	34.7	1.4	12.1	1.9	65.4	11.8	31.4	8.3
MMV665794	27.5	1.3	0.4	3.2	-1.0	1.5	-5.2	3.8
MMV665943	31.2	6.7	11.9	3.4	60.1	11.5	26.1	0.1
MMV665827	-0.4	5.4	6.0	0.5	27.6	2.6	29.0	1.1
MMV665785	0.8	1.2	0.6	5.6	14.3	9.9	8.7	3.8
MMV665971	4.7	8.4	2.8	6.1	3.1	12.6	2.7	9.4
MMV000442	2.8	2.4	-3.0	3.7	26.8	9.2	3.9	20.5
MMV007127	4.5	4.3	-5.7	0.7	37.1	8.5	28.4	7.6
MMV667486	-1.6	0.6	0.2	7.6	2.7	8.8	10.4	17.1
MMV665977	12.3	1.2	13.5	2.0	25.9	11.1	34.3	6.8
MMV020492	0.1	0.5	3.7	5.9	-1.9	7.7	5.3	2.4
MMV000648	16.8	7.1	-1.1	3.7	26.8	0.8	-4.3	8.2
MMV007654	1.4	5.5	4.4	8.4	9.8	13.9	12.2	9.2
MMV001255	1.6	1.8	4.6	3.8	14.4	12.8	13.5	11.3
methylene blue	103.2	0.4	104.0	1.2	108.7	1.7	107.2	1.7

* compounds underwent an additional freeze/thaw cycle.

Supplementary Figure S7

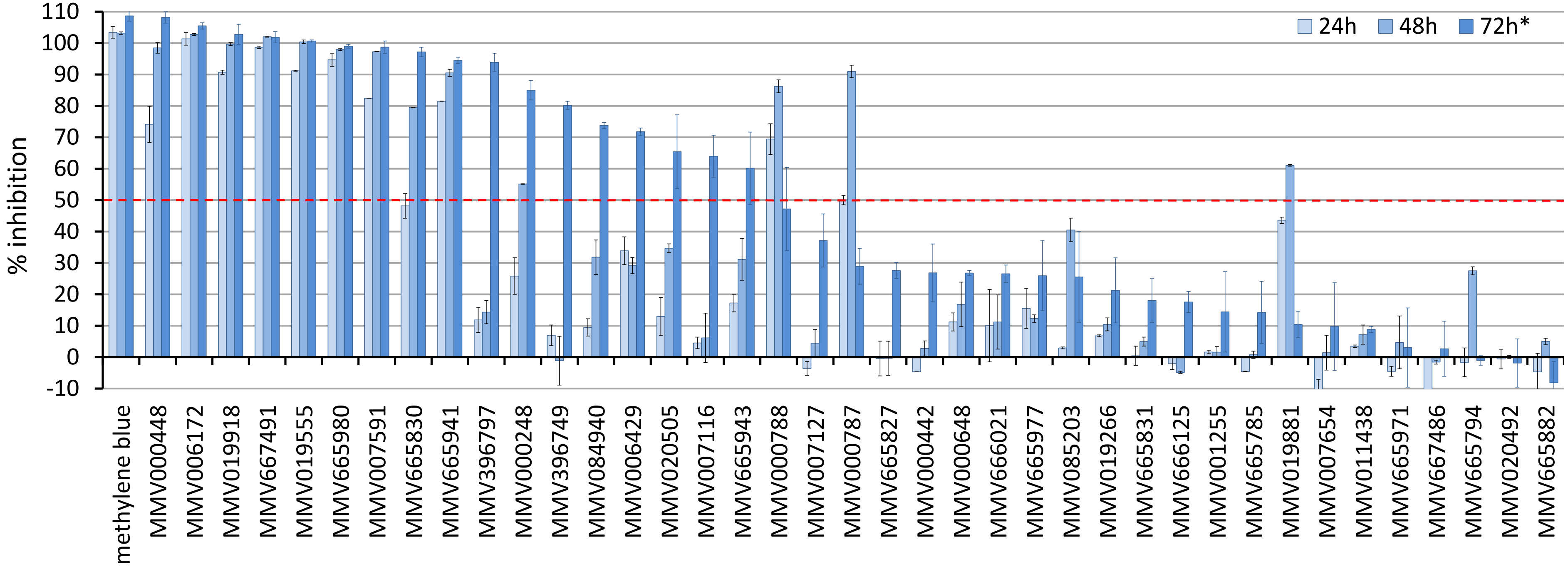

Supplementary Figure S7. Effect of incubation time on the activity of compounds in the AO-GMT assay at 24h, 48h and 72h incubation. All the compounds activity thresholds are set at 50% inhibition. * indicates an additional freeze/thaw cycle of test compounds.

Supplementary Table S5. Effect of incubation time on the potency of the GCS compounds in the AO-GMT assay.

Compound	24h		48h		72h*	
	IC_{50} (nM)	± SEM	IC_{50} (nM)	± SEM	IC_{50} (nM)	± SEM
MMV000248					~5532	
MMV000448	~1921		2077.5	439.7	1540.9	94.6
MMV000787			~3602			
MMV000788	~4991		2168.9	77.6		
MMV006172	1451.3	164.2	1044.1	73.0	632.0	33.8
MMV006429					~1784	
MMV007116					~1920	
MMV007591	~4172		1201.8	164.7	868.5	20.1
MMV019555	3924.7	71.6	3141.9	2.2	2457.2	132.1
MMV019881			~3523			
MMV019918	~3177		1103.3	343.4	1006.0	35.5
MMV020505					~7665	
MMV084940					~5750	
MMV396749					~2321	
MMV396797					~4971	
MMV665830			~6027		~3865	
MMV665941	~5279		~2969		2073.4	98.7
MMV665943					~5688	
MMV665980	836.3	124.8	425.0	44.4	630.0	41.4
MMV667491	~5224		~4367		2740.2	71.4
methylene blue	1356.7	246.1	241.6	4.1	226.0	59.2

* indicates an additional freeze/thaw cycle of test compounds.

Missing values correspond to compounds/time points for which a 4-parameter logistic curve could not be fitted (inactives). An estimation of the IC_{50} value is given for curves that did not reach an inhibition plateau.

Supplementary Figure S8

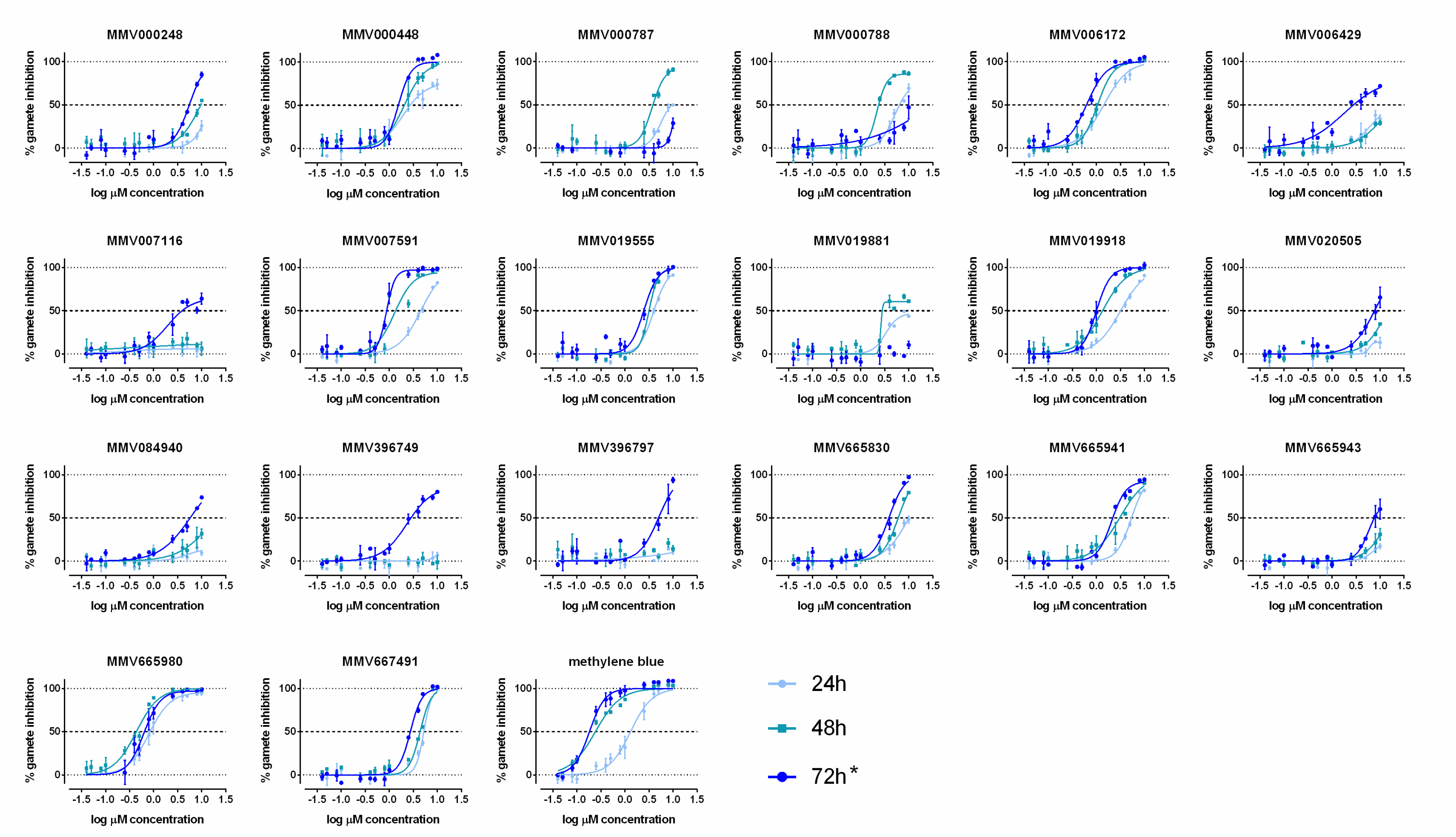

Supplementary Figure S8. Effect of incubation time on the potency of compounds in the AO-GMT assay at 24h, 48h and 72h incubation. * indicates an additional freeze/thaw cycle of test compounds.
